# Location and Extent of Paravascular Nerve Fiber Layer Clefts in Eyes with Epiretinal Membranes

**DOI:** 10.3390/jcm13195731

**Published:** 2024-09-26

**Authors:** Sekita Dalsgård Petersen, Ulrik Correll Christensen, Michael Larsen

**Affiliations:** 1 Faculty of Health and Medicine, University of Copenhagen, 2200 Copenhagen, Denmark; 2 Department of Ophthalmology, Rigshospitalet, 2600 Glostrup, Denmark

**Keywords:** paravascular nerve fiber layer clefts, epiretinal membrane, *en face* optical coherence tomography, inner limiting membrane

## Abstract

**Purpose:** The clinical use of *en face* optical coherence tomography (OCT) has revealed nerve fiber layer clefts in the retinal nerve fibers in eyes with macula-centered epiretinal membranes (ERMs). The purpose of this study is to describe the location and the extent of retinal nerve fiber layer (RNFL) clefts in eyes with symptomatic ERMs. **Methods:** We conducted a retrospective review of 17 individual eyes in 17 patients with symptomatic ERMs and a control group of 10 healthy eyes from 10 subjects who had been examined for unrelated causes. The examinations performed included best-corrected visual acuity, rebound tonometry, fundus photography, structural OCT and angiographic OCT (OCTA) made in the form of 12 × 12 mm angiographic volume scans. **Results:** Hyporeflective RNFL clefts, seen in 14 out of 17 eyes with ERMs, were sharply demarcated in the *en face* presentation of slabs extending from the internal limiting membrane through the RNFL or including only the latter. The clefts were capillary-free on OCTA scans and formed depressions of the retinal surface. Most of the clefts were adjacent to and followed the course of the retinal trunk vessels, but clefts were also seen along smaller macular vessels and beyond the retinal vascular arcades. **Conclusions**: Paravascular RNFL clefts can be observed beyond the vascular arcades and adjacent to small vessels on OCTA block scan data. This suggests that the direction and magnitude of tractional displacement of the inner retina in eyes with epimacular membranes can extend beyond the vascular arcades and add to an improved analysis of abnormal fundus findings.

## 1. Introduction

Epiretinal membranes (ERMs) are a relatively common finding in adults and can be observed in approximately 6–34% of people in a population above the age of 40 [1,2,3]. ERMs are mainly found in the macula, but they can extend beyond the vascular arcades of the retina [4]. The prevalence of ERMs in men and women is comparable, and the frequency of ERMs in right and left eyes is the same [1,3]. In most cases, patients have mild or no visual impairment, but the membrane can slowly expand and thicken while increasing its tractional deformation of the retina and causing impaired vision [4,5,6].

In this study, we report the location and extent of retinal nerve fiber layer (RNFL) clefts. They possess characteristics known by many names in previous studies, e.g., cleavage, inner retinal defects and paravascular abnormalities. On cross-sectional OCT scans, RNFL clefts present as a depression of the inner retinal surface. They have also been observed on fundus photography, the first time in eyes with high myopia and, later, in non-myopic eyes and studies related to ERMs on fundus photography, as well as cross-sectional and *en face* OCT [7,8,9,10,11,12]. The clefts are faintly dark on fundus photography, but they stand out with great contrast in optical coherence tomography (OCT), especially on the *en face* presentations of the RNFL, and they have been seen to shrink after surgical ERM removal [7,9]. They have been observed along the vascular arcades and major vessels, but, to the best of our knowledge, no studies have described RNFL clefts or similar changes observed along small vessels and beyond the vascular arcades [7,10,13]. Previous studies have suggested that the traction of the ERM causes RNFL clefts [10].

It is not clear whether RNFL clefts contribute to symptomatic vision loss. Previous studies found RNFL clefts in asymptomatic eyes and cystoid or fissure-like spaces in healthy eyes but no abnormal visual field test corresponding to RNFL clefts [12,13,14]. A few studies reported reduced retinal sensitivity measured with microperimetry that corresponded to the field of RNFL clefts [9,15].

Cystoid or fissure-like spaces adjacent to the temporal arcade vessels and most often with no connection to the vitreous cavity on cross-sectional OCT scans have been observed in healthy eyes [14]. These structures cannot be observed on fundus photography and have not been reported on OCTA or structural OCT. The broad typical clefts of the inner retinal layers that can be observed on OCTA scans, structural OCT scans and sometimes on fundus photography have not been observed in healthy, non-myopic eyes.

This report describes a study of the location and extent of RNFL clefts in eyes with symptomatic macular ERMs that had been referred for surgery using dense wide-field OCT volume scans that were rendered in structural and angiographic modes as deemed optimal for the analysis of anatomical relations. The results show that OCTA block scan data contain information that, if properly extracted and presented, provides valuable insight into the tractional effects of RNFL clefts, which often extend beyond the vascular arcades. We think that the evidence related to the peripheral and widespread location of clefts reported in this study contributes to an improved analysis and less misinterpretation of abnormal fundus findings.

## 2. Methods

This exploratory retrospective study included 17 individual eyes in 17 patients repeatedly referred for preoperative evaluations of ERM retinopathy with associated visual complaints within a two-week period. The exclusion criteria were high myopia (−6D or greater) and glaucoma in evaluated eyes. The eye examinations included best-corrected visual acuity measured on an electronic Snellen chart, rebound tonometry (iCare, Icare Finland Oy, Vantaa, Finland), fundus photography and 12 × 12 mm structural OCT and angiographic OCT (OCTA) volume scans (Topcon DRI OCT Triton, Topcon, Tokyo, Japan). The inner retinal layers were analyzed using the *en face* viewing of automatically generated slabs, ranging from the inner limiting membrane through the RNFL/ganglion cell layer (IMAGEnet 6 version 1.31.18920, Topcon, Tokyo, Japan). The patients also reported a degree of preoperative metamorphopsia that was categorized as nonexistent, marginal, moderate, or extensive by the clinician. A clinical evaluation found 13 eyes eligible for surgery, and 12 were operated on. Preoperative OCT scans were repeated at the postoperative visit in a subset of the studied eyes. The patients’ other eyes were not included as internal controls because several patients, on examination, were found to have ERM on the other eye also and/or reduced BCVA on the other eye. The control group was composed of 10 heathy eyes in 10 subjects. The 10 subjects included for comparison had been examined in the emergency department for unrelated causes, including retinal tears, vitreous hemorrhage, amotio, chemical eye injuries and acute glaucoma in one eye. They were only included in the control group if the condition they had been examined for was in one eye only. The other eye, which was included in the control group, had to be healthy with no history of eye disease. Conditions such as cataract and refraction errors of +/− 6D on examination were accepted and was not an exclusion criteria in the control group. The eye examinations in the control group also included BCVA, rebound tonometry and the same types of OCT scans. Comparison of the ERM group and control group is presented in Table 1.

The OCT scans of the patients were analyzed and categorized in groups with RNFL clefts along the vascular arcades, along small vessels and beyond the vascular arcades. The first group was characterized by RNFL clefts adjacent to the vascular arcades with no clear inner retinal tissue between the vessel and the cleft on structural OCT and OCTA scans. The second group was characterized on OCTA scans by RNFL clefts along small vessels with no clear inner retinal tissue between the cleft and the small vessel. The clefts in this group were identified on the *en face* view of the structural OCT slab, because they were clearer on this scan. Cleft locations along vessels were also verified via detection on OCTA scans. The third group was characterized by clefts located on the periphery of the vascular arcades.

The examinations were performed from 2020 to 2023. All procedures adhered to the standards of the Declaration of Helsinki. The participants provided written informed consent to the publication of their data.

## 3. Results

### 3.1. Review of Healthy Eyes

**Figure 1:**
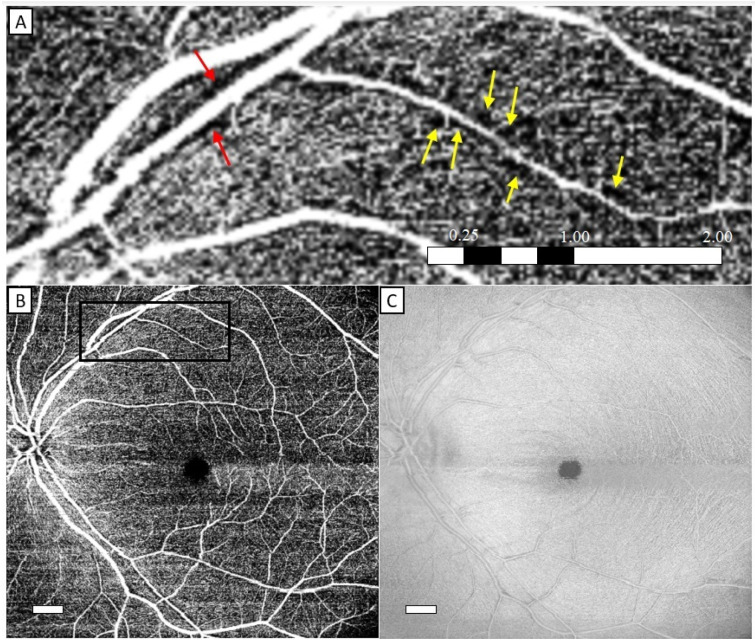
A healthy eye with perivascular capillary-free zones, as depicted by OCTA, around a large retinal arteriole ((**A**), red arrows) and a smaller retinal arteriole ((**A**), yellow arrows) along the upper vascular arcade (**B**), with the box indicating the location of the magnified view. *En face* structural OCT view (**C**) of intact inner retinal layers with vessels that display a central hyperreflective reflex with accompanying hyporeflective intravascular layers. The white boxes in (**B**,**C**) are scalebars with a length corresponding to 1 mm. The length of the scalebar in (**A**) is in mm.

Our review of the *en face* structural OCT and OCTA slabs of the healthy eyes showed no RNFL clefts and no unexpected findings, and the periarterial capillary-free zones were of a width corresponding to measurements made with OCTA and adaptive optics scanning laser ophthalmoscopy (AOSLO), i.e., approximately 67 μm (Figure 1) [16]. Prior studies have measured a periarterial capillary-free zone of 30–50 μm in histological tissue preparations [17,18,19,20]. Most of the healthy eyes showed areas adjacent to vessels that were slightly hyporeflective on the *en face* view of the structural OCT slab. On cross-sectional scans, the slightly hyporeflective areas occasionally corresponded to cystoid spaces. The *en face* view of the structural OCT slab did not show the characteristic hyporeflective, broad and demarcated RNFL clefts as seen in the ERM group.

The healthy eyes had spherical refractive errors of +/− 6D. All had intraocular pressure of 21 mmHg or lower. All had a BCVA of 1.0 or above.

### 3.2. Review of Eyes with ERM

**Figure 2:**
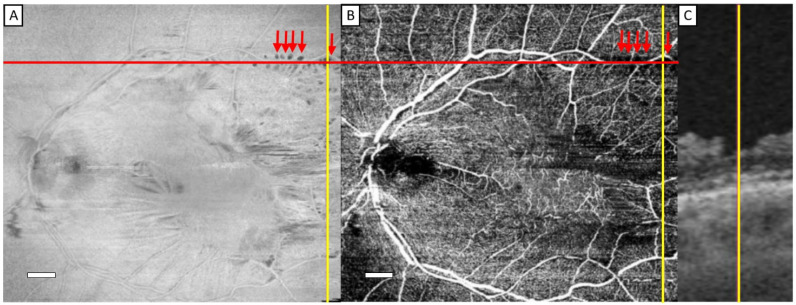
Representative examples of nerve fiber layer clefts in patient No. 7. *En face* view of the structural OCT slab (**A**) with several hyporeflective nerve fiber layer clefts in a regular pattern adjacent to the vascular arcades (red arrows). An angiographic OCT slab (**B**) with capillary-free zones (red arrows) corresponding to the nerve fiber layer clefts on *en face* view of the structural OCT slab. The length of the white scalebar in (**A**,**B**) is 1 mm. A structural cross-sectional scan (**C**) showing a depression corresponding to the nerve fiber layer cleft on *en face* view of the structural OCT and OCTA slabs. The cross between the yellow and red line in (**A**,**B**) shows the location of the cross-sectional scan. The width of the cleft in (**C**) is approximately 0.2 mm.

**Figure 3:**
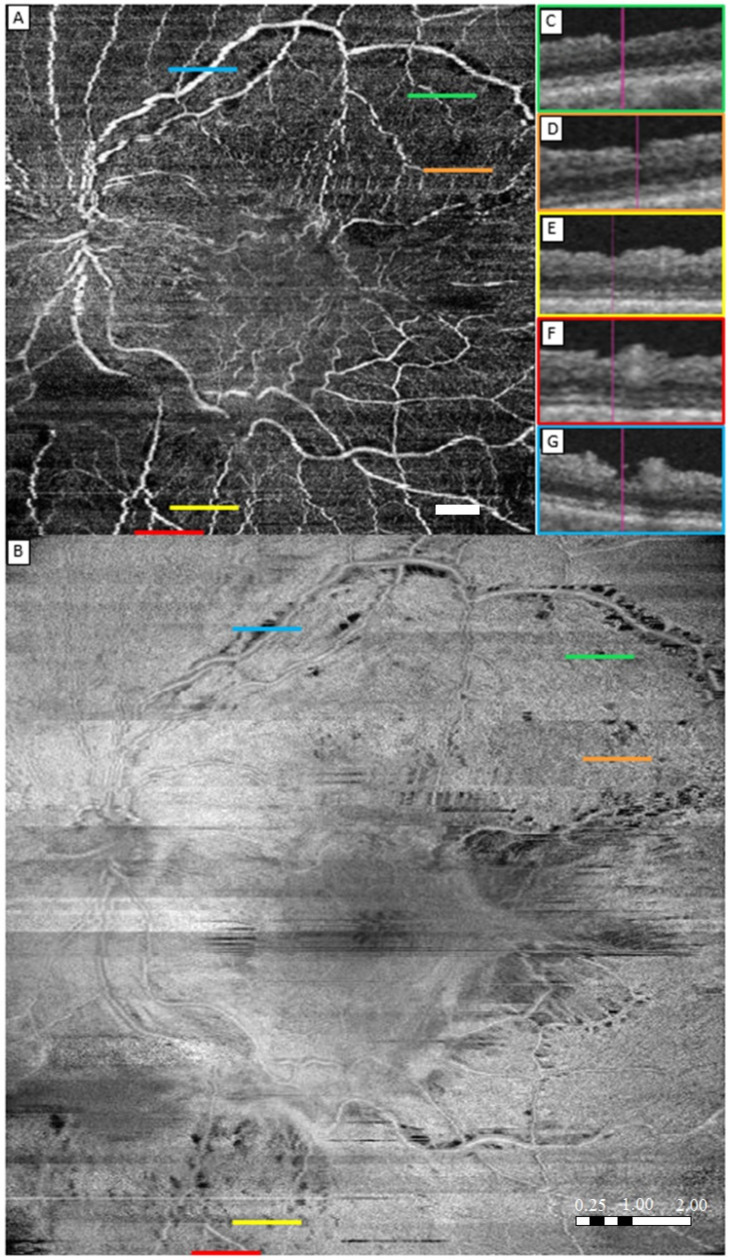
Representative examples of nerve fiber layer clefts in patient No. 6. OCTA slab (**A**), and *en face* view of the structural OCT slab (**B**) showing an eye with ERMs with nerve fiber layer clefts along the major retinal vessels. Each colored line in (**A**,**B**) indicates the location of the cross-sectional OCT slab with the corresponding color. (**C**,**D**) show depressions on cross-sectional OCT scan corresponding to a nerve fiber layer cleft on *en face* view of the structural OCT slab and capillary-free area on OCTA slab. On the *en face* view of the structural OCT slab, the lesion does not seem to be adjacent to a vessel, but the OCTA reveals that it is placed near a small vessel. (**E**) shows a depression on a cross-sectional OCT slab similar to the clefts described above. However, the OCTA and *en face* view of the structural OCT slab reveal that the cleft is beyond the vascular arcades. (**F**) shows a similar cleft that is barely captured by the scan. (**G**) shows a typical cleft along the major retinal vessels. The length of the white scalebar in (**A**) is 1 mm. The big white scalebar in (**B**) shows a length of 1 mm, and the small black and white bars show a length of 0.25 mm each. The clefts in C-G are approximately 60–200 μm on the cross-sectional scans.

Our review of the *en face* view of the structural OCT slabs of ERM eyes showed hyporeflective RNFL clefts, sharply demarked, most often adjacent to and following the course of the major vessels of the vascular arcades and rarely adjacent to smaller vessels around the macula (Figure 2 and Figure 3 and Table 2). The hyporeflective RNFL clefts on the *en face* view of the structural OCT slab appeared as dark capillary-free zones on OCTA slabs. A cross-sectional view of the structural OCT scan typically showed a depression of the retinal surface corresponding to the hyporeflective RNFL clefts. On fundus photography, only a fraction of the clefts were seen as dark areas. Fourteen patients clearly exhibited RNFL clefts. They were present in the macular area, as well as in peripheral areas near the vascular arcades, and sometimes beyond the vascular arcades (Figure 3 and Table 2). The prevalence of RNFL clefts along the vascular arcades, along small vessels and beyond the vascular arcades is shown in Table 2 and Table 3. The first was more common than the latter two. Table 2 and Table 3 show that RNFL clefts along small vessels and beyond the vascular arcades only occur in eyes with RNFL clefts along the vascular arcades.

Forty percent of patients with RNFL clefts beyond the vascular arcades had extensive metamorphopsia. Twenty-nine percent of patients with RNFL along the vascular arcades only had extensive metamorphopsia.

**Figure 4:**
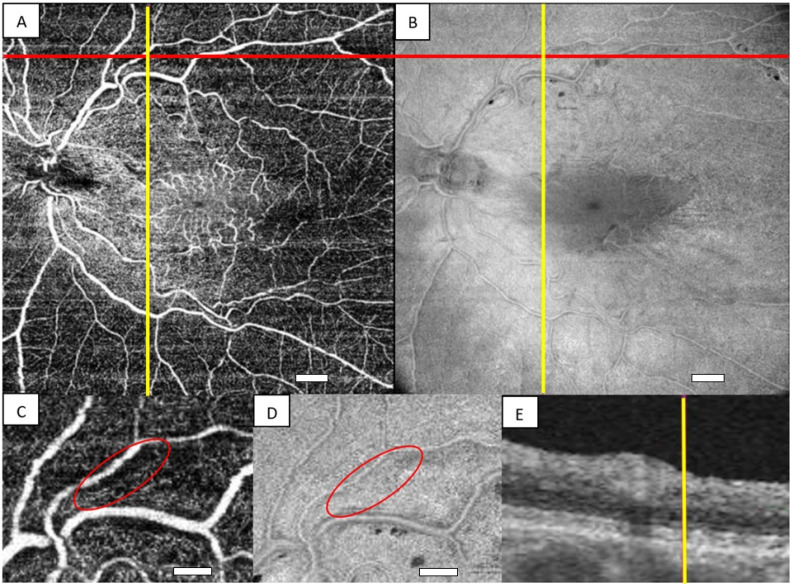
Illustration of a lesion that is capillary-free on OCTA with no corresponding hyporeflective nerve fiber layer cleft on en face view of the structural OCT slab. The cross between the red and yellow lines on OCTA (**A**) is placed on the capillary-free zone. The magnified view in (**C**) shows the section with the capillary-free zone (red ring). The cross between the red and yellow line on *en face* view of the structural OCT slab (**B**) is placed on the area corresponding to the capillary-free zone on OCTA. The magnified view in (**D**) shows the area corresponding to the capillary-free zone on OCTA (red ring). The area is not hyporeflective on *en face* view of the structural OCT slab. The red line in (**A**,**B**) indicates the orientation of the cross-sectional OCT (**E**). The yellow line in (**E**) is placed in the area that is capillary-free on OCTA. No depression is seen at the yellow line corresponding to the capillary-free zone. The length of the white scale bars in (**A**,**B**) corresponds to 1 mm. The scale bars in (**C**,**D**) correspond to 0.5 mm.

The hyporeflective RNFL clefts most often presented along the vascular arcades (Figure 2). The clefts were most prominent on *en face* OCT and least prominent on fundus photography. In four cases, the clefts also presented away from the major retinal vessels (Figure 3C,D). OCTA revealed that the clefts were adjacent to small vessels (Figure 3A). RNFL clefts were also seen beyond the vascular arcades (Figure 3E,F). Not all capillary-free areas on OCTA corresponded to a hyporeflective cleft on the *en face* view of the structural OCT slab (Figure 4).

The 17 patients included in the analysis had spherical refractive errors of +/− 6D. All had intraocular pressure of 21 mmHg or lower. Their BCVA values were between 0.05 and 0.79.

## 4. Discussion

This study supports prior analyses of extramacular characteristics of the ERM–retina complex in eyes with symptomatic visual dysfunction [6,21,22,23]. Prior studies of RNFL clefts have described clefts along the vascular arcades and major vessels [7,10,13]. In this study, RNFL clefts were found to extend beyond the retinal vascular arcades on wide-field *en face* structural OCT and OCTA scans, whereas no nerve fiber layer clefts were found in the control group’s eyes. This suggests that the tugging effect of ERMs is more peripheral than previously reported [8,9,23,24,25,26]. This study describes RNFL clefts along small vessels and beyond the vascular arcades in patients with symptomatic ERM and compares the 12 × 12 mm angiographic and *en face* view of the structural OCT slabs, extending from the inner limiting membrane through the RNFL, with fundus photography and cross-sectional OCT.

The hyporeflective RNFL clefts on *en face* structural OCT slabs are dark on OCTA and devoid of capillaries. On cross-sectional OCT, the RNFL clefts are seen as depressions on the surface of the retina. They are usually described adjacent to major retinal vessels [10,27]. In this study, we found similar abnormalities adjacent to small retinal vessels. Thus, ERMs can be seen to affect the retina more extensively than previously reported. Our observations indicate that the ERM exerts traction that spreads diffusely over a very wide area and extends beyond the vascular arcades, up to and presumably beyond the edge of the 12 × 12 mm scan.

RNFL clefts along small vessels and beyond the vascular arcades were only seen when RNFL clefts along the vascular arcades were present. Whether the clefts are helpful in the evaluation of the indication for surgery requires further study. The clefts are most prominent on the *en face* view of structural OCT slabs and can be challenging to detect on fundus photography. The relation between the clefts and blood vessels was particularly evident on OCTA, and the capillary-free zones on OCTA did not invariably correspond to depressions on cross-sectional structural OCT slabs. OCT adds important information to fluorescein angiography in that it shows vascular voids to be tissue produced by nerve fiber bundle displacement without substance loss, rather than a product of vascular obliteration [28].

Healthy vascular beds, including that of the retina, have a capillary-free zone alongside arterioles, presumably because diffusion of oxygen out of the arteriole is sufficient for the stroma. A similar but narrower zone is seen along venules [16,29,30]. Second-order arteries have the most prominent periarterial capillary-free zone [31]. Perivenular capillary-free zones increase with the distance to the fovea and the diameter of venules [16]. Narrow traction-induced clefts can be difficult to distinguish from the physiological capillary-free zone, because the clefts tend to also be paravascular, but a distinguishing feature is that the clefts are often spindle-shaped, interrupted by vascular bifurcations [9,12]. Furthermore, the perivascular capillary-free zone is not accompanied by a depression of the retinal surface [16]. A further distinction is that the perivascular capillary-free zone is wider along arteries than veins, whereas traction clefts related to ERMs and myopia tend to be wider along veins than arteries [10,16]. Vitreomacular traction is strong along the vascular arcades, which might explain why tractional nerve fiber layer clefts are commonly seen along the arcade vessels [32,33,34]. The correlation between clefts and tractional ERMs or strong vitreoretinal adhesion is supported by the finding of retinal pits adjacent to vessels in eyes with posterior vitreous detachment. Retinal pits are small defects of the inner retina which lack varying amounts of inner retinal layers and the inner limiting membrane. They are only seen in areas with posterior vitreous detachment [35]. Furthermore, clinical evidence of strong vitreoretinal traction along the vascular arcades is present during surgery with inner limiting membrane peeling. Vitreoretinal traction can be noticed when reaching the vascular arcades as it becomes challenging to peel the membrane, and it is only rarely possible to continue peeling the membrane across the vascular arcades.

Our study showed that most healthy eyes in the control group had slightly hyporeflective areas adjacent to vessels on the *en face* view of the structural OCT slab, and some of them corresponded to cystoid spaces on cross-sectional scans similar to the cystoid or fissure-like spaces describes in healthy eyes in previous reports [14]. None of the healthy eyes exhibited the characteristic hyporeflective, broad and demarcated RNFL clefts on the *en face* view of the structural OCT slab. The typical broad RNFL clefts have not been observed in healthy eyes in previous studies. We therefore hypothesize that some RNFL clefts observed in this study might appear when the traction of the ERM stretches lesions that might already exist in healthy eyes.

Macular hole and ERM surgery with inner limiting membrane peeling can induce dissociation of nerve fiber layer bundles, which is seen as dark curved areas on fundus photographs, dark dots along the nerve fiber layer on *en face* OCT, capillary voids on OCTA and retinal depressions on OCT that follow the nerve fibers instead of the vessels [36,37,38,39]. It has been suggested that ERMs can induce inner limiting membrane defects that induce nerve fiber dissociation [40,41]. The observations made here and in certain prior studies show that in eyes with ERMs, DONFL may be present before the surgical peeling of the inner limiting membrane [40].

Miyoshi et al. described perivascular inner retinal defects in eyes with idiopathic ERMs. Their study showed that 38.3% of their patients had perivascular inner retinal defects [27]. In our study, 82% had similar defects, perhaps because our sample consisted only of symptomatic eyes.

Abnormal fundus findings including RNFL clefts can be challenging to categorize and can be misinterpreted, e.g., as RNFL defects associated with glaucoma or periarterial or venular capillary-free zones [7,10,15,42,43]. This study adds to a more precise assessment of abnormal fundus findings.

Our study showed that 40% of patients with RNFL clefts beyond the vascular arcades had extensive metamorphopsia and 29% of patients with RNFL clefts along the vascular arcades only had extensive metamorphopsia. Whether the extent of RNFL clefts is valuable in preoperative assessments requires further studies.

This study is limited by the sample size, the breadth of variation in severity and the size of the OCT scans. Further studies are required to conclude if RNFL clefts can be observed more peripherally than the area in 12 × 12 mm OCT scans observed in this study.

## 5. Conclusions

In conclusion, we report the existence of paravascular RNFL clefts beyond the vascular arcades and adjacent to small vessels in symptomatic eyes with ERM. This indicates that the way the ERM affects the retina is more extensive and reaches further away from the macula than previously described. It is not clear whether this phenomenon has any clinical consequences, but the insight is of value in the analysis of abnormal fundus findings in clinical practice.

## Figures and Tables

**Table 1:** Comparison of the ERM group and control group.

	ERM Group	Control Group
Mean age	68 (range 59–76)	65 (range 58–73)
Right eyes	3	7
Left eyes	14	3
Females	9	4
Males	8	6

**Table 2:** Patient data showing each patient’s characteristics, the location of the nerve fiber layer clefts in each patient and their medical history of metamorphopsia.

Patient Number	Age	Sex	Eye	Nerve Fiber Layer Clefts			Metamorphopsia
				Along the Vascular Arcades	Along Small Vessels	Beyond the Vascular Arcades	
1	64	Female	Left	Yes	No	No	Yes—extensively
2	71	Female	Left	Yes	No	Yes	No
3	63	Male	Left	No	No	No	Yes—extensively
4	66	Female	Left	Yes	Yes	No	No
5	74	Male	Left	Yes	No	No	No
6	66	Female	Left	Yes	Yes	Yes	Yes—extensively
7	72	Male	Left	Yes	Yes	No	Yes—extensively
8	69	Male	Left	No	No	No	Yes—marginally
9	62	Female	Left	Yes	No	No	Yes—extensively
10	65	Female	Right	Yes	No	No	No
11	59	Female	Left	Yes	No	Yes	No
12	76	Male	Left	No	No	No	Yes—extensively
13	69	Female	Left	Yes	No	No	No
14	68	Male	Left	Yes	No	No	No
15	71	Male	Left	Yes	No	Yes	Yes—marginally
16	66	Male	Right	Yes	Yes	Yes	Yes—extensively
17	70	Female	Right	Yes	No	No	Yes—moderately

**Table 3:** Showing the number of eyes with nerve fiber layer clefts and the location of the clefts.

Nerve Fiber Layer Clefts	Along the Vascular Arcades	Along the Vascular Arcades + Along Small Vessels	Along the Vascular Arcades + Beyond the Vascular Arcades	Along the Vascular Arcades + Along Small Vessels + Beyond the Vascular Arcades
No. of eyes	14	4	5	2

## Data Availability

The datasets presented in this article are not readily available because of limitations imposed by the General Data Protection of the European Union. Requests to M.L.
